# Harnessing the CRISPR-Cas Systems to Combat Antimicrobial Resistance

**DOI:** 10.3389/fmicb.2021.716064

**Published:** 2021-08-20

**Authors:** Cheng Duan, Huiluo Cao, Lian-Hui Zhang, Zeling Xu

**Affiliations:** ^1^Integrative Microbiology Research Center, South China Agricultural University, Guangzhou, China; ^2^Department of Microbiology, Li Ka Shing Faculty of Medicine, The University of Hong Kong, Pokfulam, Hong Kong SAR, China

**Keywords:** CRISPR-Cas system, antimicrobial resistance, phage delivery, plasmid curing, genome targeting

## Abstract

The emergence of antimicrobial-resistant (AMR) bacteria has become one of the most serious threats to global health, necessitating the development of novel antimicrobial strategies. CRISPR (clustered regularly interspaced short palindromic repeats)-Cas (CRISPR-associated) system, known as a bacterial adaptive immune system, can be repurposed to selectively target and destruct bacterial genomes other than invasive genetic elements. Thus, the CRISPR-Cas system offers an attractive option for the development of the next-generation antimicrobials to combat infectious diseases especially those caused by AMR pathogens. However, the application of CRISPR-Cas antimicrobials remains at a very preliminary stage and numerous obstacles await to be solved. In this mini-review, we summarize the development of using type I, type II, and type VI CRISPR-Cas antimicrobials to eradicate AMR pathogens and plasmids in the past a few years. We also discuss the most common challenges in applying CRISPR-Cas antimicrobials and potential solutions to overcome them.

## Introduction

**Figure 1:**
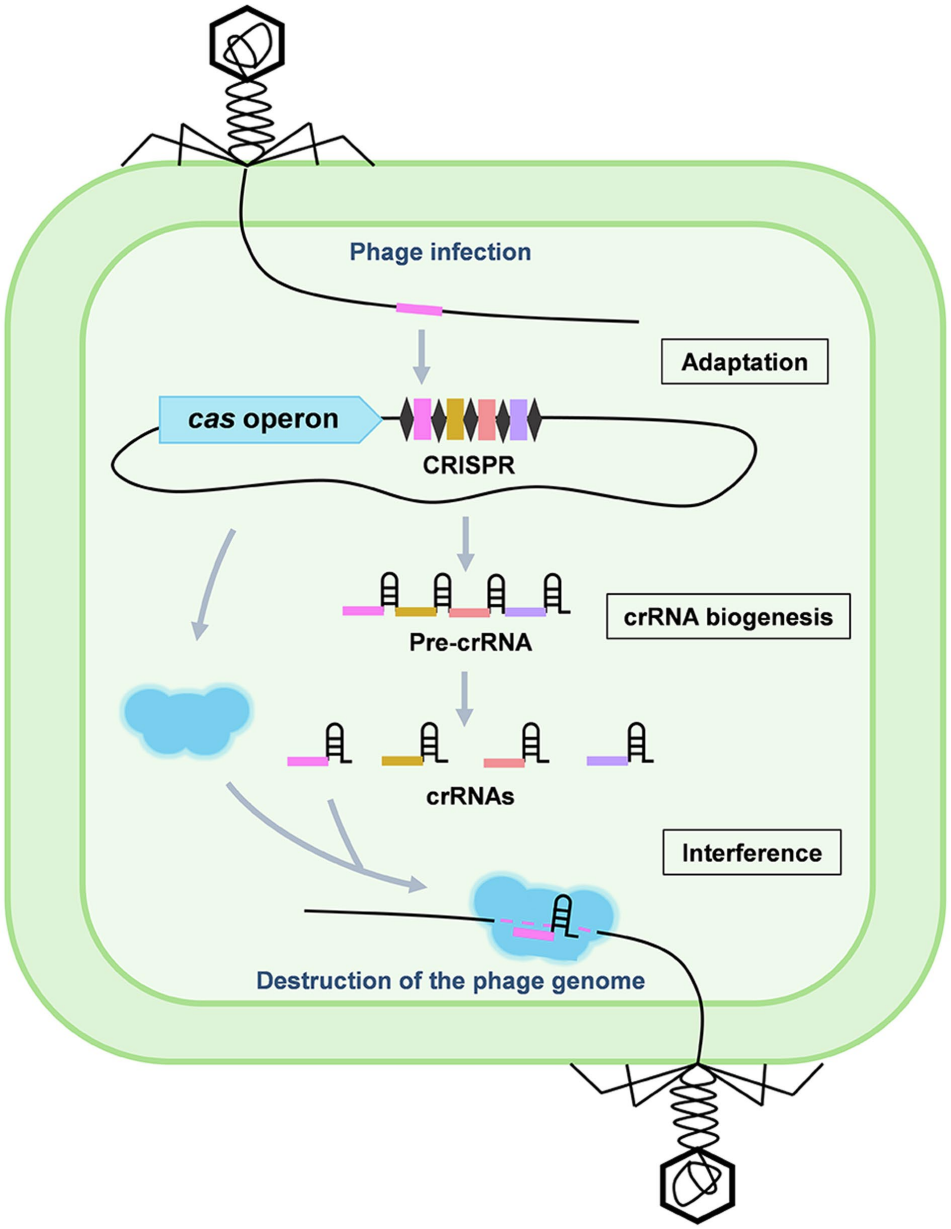
Stages of the CRISPR-Cas adaptive immunity. CRISPR-Cas immunity consists of three stages: adaptation, crRNA biogenesis, and interference. During adaptation, a DNA fragment of the invading genetic elements, such as a phage genome, is captured and incorporated into the CRISPR array to generate a new spacer (pink). During crRNA biogenesis, the entire CRISPR array is transcribed into a long pre-crRNA which is further processed into mature crRNAs. During interference, the crRNA specifically recognizes a target protospacer sequence in the invaders by base pairing and guides the Cas effector to destruct the targets.

CRISPR-Cas system has been identified as an adaptive immune system which enables prokaryotes to resist invading genetic elements (basically viruses and plasmids) through foreign DNA/RNA destruction (Marraffini, 2015). Generally, a CRISPR-Cas system is organized with a CRISPR locus and its accompanying *cas* operon, performing immunity in three stages: adaptation, CRISPR RNA (crRNA) biogenesis and interference (Figure 1; Nussenzweig and Marraffini, 2020). According to their differences in the complexity of effecter modules, CRISPR-Cas systems are grouped into two classes (1 and 2; Makarova et al., 2020). The class 1 systems, including the type I, III, and IV systems, are characterized by using a multi-subunit effector complex in combination with an additional Cas nuclease to destruct the target nucleic acids, while the class 2 type II, V, and VI systems utilize a single multi-domain effector to execute target destruction. Owing to the great programmability of CRISPR-Cas systems, in the past decade, they have largely elevated our ability to detect, destruct, manipulate, and annotate specific nucleic acids sequences in diverse living organisms, revolutionized the field of genetics (Pickar-Oliver and Gersbach, 2019).

Antimicrobial resistance is posing a serious threat to the global health. It was estimated that approximately 10 million people will die annually suffering from AMR (Antimicrobial-resistant) pathogens if no action is taken now (de Kraker et al., 2016). AMR pathogens are typically equipped with complicated intrinsic and adaptive resistant mechanisms as well as abilities to easily acquire transmissible AMR genes especially in the plasmid-mediated manner from the environment (Munita Jose et al., 2016), which confer to these pathogens great resistant capacity to survive and thrive from routine antimicrobial chemotherapies and cause continuous infections. Due to the development of new antibiotics is far slow than the establishment of bacterial resistance to them, other novel and effective antimicrobial strategies that can take place of antibiotics are urgently required to relieve the global crisis of antimicrobial resistance. Noticeably, increasing studies have shown that CRISPR-Cas systems are emerging as one of the most promising candidates to deal with antimicrobial resistance in recent years (Bikard and Barrangou, 2017; Gholizadeh et al., 2020). In this mini-review, we summarize the recent advances in developing CRISPR-Cas antimicrobials and discuss the main challenges in practical uses as well as their potential solutions.

## Crispr-Cas-Based Antimicrobial Exploitations

**Figure 2:**
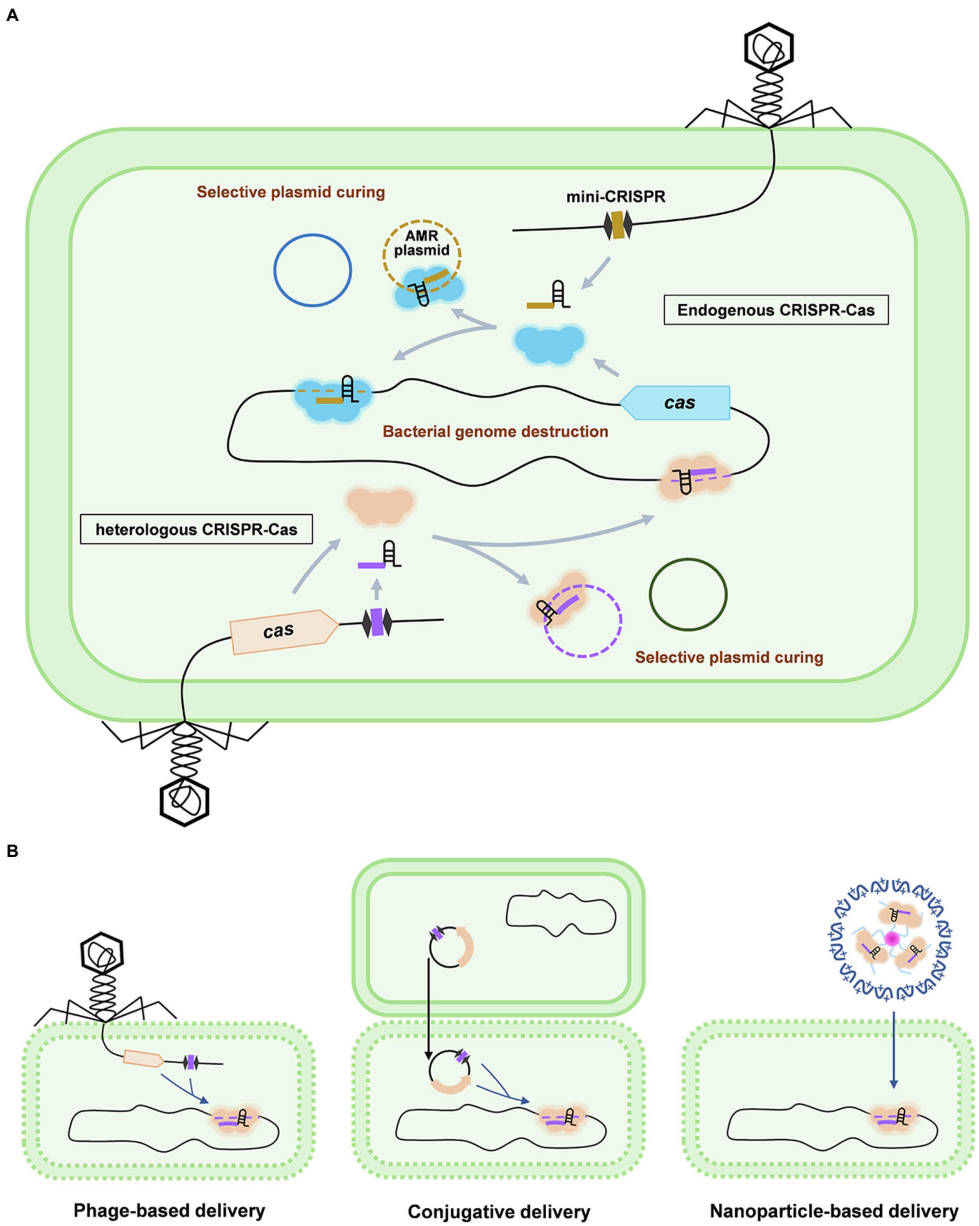
Working mechanisms and delivery of CRISPR-Cas antimicrobials. **(A)** Antimicrobial applications based on the endogenous and heterogeneous CRISPR-Cas systems are shown using the phage-based delivery as an example. In the strains containing an active CRISPR-Cas system (upper panel), a single mini-CRISPR element is required to express a crRNA to guide the endogenously expressed Cas effector to specifically destruct the host genome or cure the AMR plasmid, which results in the killing or re-sensitizing of the AMR pathogens, respectively. Mini-CRISPR and *cas* genes can also be co-delivered into the target cells to achieve bacterial genome destruction or plasmid curing (lower panel). **(B)** Delivery strategies for CRISPR-Cas antimicrobials: phage-based delivery (left), conjugative plasmid-based delivery (middle), and nanoparticle-based delivery (right).

Owing to the power of RNA-guided destruction of nucleic acids, CRISPR-Cas system becomes a promising candidate for the development of the next-generation antimicrobials to deal with infectious diseases especially those caused by AMR pathogens (Figure 2A). Moreover, the flexible programmability of the CRISPR-Cas system can selectively kill a particular bacterial member within a large population, which enables CRISPR-Cas antimicrobials to precisely modulate the composition of a complex bacterial population and will be extremely useful to treat infections within a natural complex microbial consortia, such as the gut microbiome. This is obviously superior to the conventional antibiotics which tend to be broad spectrum without killing specificity.

Based on the anticipated advantages as mentioned above, in the past decade, increasing studies were dedicated to exploiting the antimicrobial potentials of CRISPR-Cas systems and have demonstrated that intentional programming the systems to target bacterial genomes are extremely cytotoxic to bacterial cells. The first report on CRISPR-Cas-mediated bacterial killing was published by Edgar and Qimron in 2010 (Edgar and Qimron, 2010), which showed that directing the endogenous CRISPR-Cas system to an integrated prophage led to the death of 98% cells in an *Escherichia coli* population. Since most bacterial species lacks nonhomologous end joining to repair DNA damage (Donohoue et al., 2018), efficient bacterial killing caused by programmed genome targeting was demonstrated in a number of clinically relevant AMR species, such as *Clostridium* species, *E. coli*, and *Pseudomonas aeruginosa*, using either endogenously or heterologously expressed CRISPR-Cas systems (Jiang et al., 2013; Kiro et al., 2014; Pyne et al., 2016; Xu et al., 2019). However, genome targeting in these studies was employed to further achieve genome editing with the provision of DNA repair templates (Xu et al., 2021b), comparatively little attention was paid using these systems to eradicate AMR pathogens for antimicrobial applications. Here, in this section, we summarize the major achievements of developing CRISPR-Cas antimicrobials to combat antimicrobial resistance with a focus on the type I, type II, and type VI systems.

### Type I System

Type I CRISPR-Cas systems, recognized by the signature Cas3 nuclease, are the predominant systems found in prokaryotes which account for nearly 60% of all CRISPR-Cas systems identified so far (Makarova et al., 2015). One of the pioneering studies about developing CRISPR-Cas antimicrobials is based on the well-characterized type I-E CRISPR-Cas system from *E. coli*. By deleting the *hns* gene which functions as a repressor of the endogenous CRISPR-Cas system in *E. coli* (Pul et al., 2010), Gomaa et al. showed a substantially decreased cell recovery by transforming a plasmid (*α-ftsA*) encoding a crRNA targeting the essential gene *fstA in vitro* (Gomaa et al., 2014). Similar result of reduced cell recovery (> 99.999%) was observed by the introduction of the *α-ftsA* plasmid together with two additional plasmids pCasA-E and pCas3 expressing the *cas* genes, indicating the strong potency of bacterial killing using the type I-E CRISPR-Cas system. In this study, the authors also demonstrated that efficient bacterial killing can be achieved by targeting any locations in the bacterial genome including the essential genes or non-essential genes, template strand or non-template strand. Importantly, bacterial strains can be selectively removed from a mixed population of strains which share high genome homology (99%), highlighting the killing specificity of CRISPR-Cas antimicrobials.

In addition to the plasmid-based transformation of the crRNA-expressing element (mini-CRISPR), antimicrobial capability of endogenous CRISPR-Cas systems was explored in combination with phage-based delivery of mini-CRISPR. *Clostridium difficil*e is a strictly anaerobic spore-forming bacterium and a major cause of intestinal infection in individuals following antibiotic treatment and phage therapy was considered as a promising strategy to treat *C. difficil*e infection (Nale et al., 2018). However, all the isolated *C. difficil*e phages are characterized as temperate phages, which exhibit diverse mechanisms, such as the expression of superinfection exclusion proteins to cause phage resistance (Bondy-Denomy et al., 2016). Owing that nearly all sequenced *C. difficile* isolates express type I-B CRISPR-Cas systems and all of them have been indicated to be active to interfere invader DNA (Boudry et al., 2015), Selle et al. engineered a *C. difficile* phage ΦCD24-2 to carry a mini-CRISPR which was designed to target a chromosomal gene encoding RNase Y and named this phage as a CRISPR phage (Selle et al., 2020). Compared to the wild-type phage which only caused 1-log reduction in the numbers of recovered bacterial cells after 2-h incubation, the CRISPR phage displayed 3-log reduction in the numbers of recovered cells. Consistent with the increased efficacy of the CRISPR phage in killing *C. difficile in vitro*, it was much more effective than the wild-type phage when examined in a mouse infection model as well. Therefore, this study not only demonstrated that CRISPR-Cas systems can enhance the potency of phage therapy but also indicated that phages would be ideal vectors to deliver the CRISPR-Cas elements for clinical applications.

Despite the convenience of only a single mini-CRISPR is required to work with the endogenous CRISPR-Cas system for efficient and site-specific genome targeting, unfortunately, a very small portion of natural CRISPR-Cas systems have been fully characterized and most of them are found inactive in some species (van Belkum et al., 2015). Thus, in most cases, it is necessary to introduce both the mini-CRISPR and Cas nucleases into the target strains (Figure 2A). To this end, Yosef et al. incorporated a large type I-E CRISPR-Cas system (five Cascade genes with one nuclease gene *cas3*) and a mini-CRISPR into a λ phage genome by replacing the genes that are not essential for phage growth and lysogenization (Yosef et al., 2015). The mini-CRISPR was designed to target two AMR genes *ndm-1* and *ctx-M-15*. In the treatment which simulates the conditions of hospital surfaces or skin flora, *E. coli* cells lysogenized with the engineered λ phage efficiently eradicate the corresponding AMR plasmids.

### Type II System

In contrast to the type I CRISPR-Cas system which requires a Cascade complex and an additional Cas3 nuclease to achieve interference on the target sequence, type II system represents a more portable system with the great simplicity of only requiring a single effector protein. Therefore, the type II system should be easier being incorporated into the phage genome for delivery compared to the type I systems. So far, applications of the type II systems have been extensively explored in the pathogenic strains, such as the Gram-negative *E. coli* and Gram-positive *Staphylococcus aureus* strains (Bikard et al., 2014; Citorik et al., 2014; Kim et al., 2016; Park et al., 2017; Rodrigues et al., 2019; Wang et al., 2019). In 2014, two elegant studies independently reported that the CRISPR-Cas9 system, which is the most common and well-characterized type II system, can be programmed to selectively kill the AMR pathogens *E. coli* and *S. aureus* (Bikard et al., 2014; Citorik et al., 2014). Both studies demonstrated that introduction of a plasmid-borne Cas9 and a mini-CRISPR into the bacterial cells containing target AMR genes on their chromosome led to a significantly decreased transformation efficiency relative to the cells lacking the target genes. Then, they explored the use of phagemids to deliver the components of Cas9 and mini-CRISPR, which demonstrated that CRISPR-Cas9 antimicrobials can not only selectively eradicate the AMR pathogen in a mixed population but also cure the AMR plasmids in particular strains. Moreover, efficacies of the CRISPR-Cas9 antimicrobials were assessed by examining their abilities of killing the enterohemorrhagic *E. coli* in *Galleria mellonella* larva and *S. aureus* on the skin of mice, respectively, showing great potentials of these systems in clinical therapies (Bikard et al., 2014; Citorik et al., 2014).

Mutations of AMR genes are ubiquitous in nature. For instance, a group of β-lactamases containing over 1,000 variants were reported which are collectively named as extended-spectrum β-lactamases (ESBLs; Paterson and Bonomo, 2005; Kim et al., 2016). As we know, Cas effectors rely on a guide crRNA for specific interference and the crRNA is typically 20~40bp in size, indicating that a crRNA designed based on one variant from a group of AMR gene might be not effective to target other variants within the same group. Taking the ESBLs as an example, a further study was conducted to search for a conserved sequence within more than 1,000 ESBL members and designed a universal crRNA to target this common β-lactamases gene group in *E. coli*, expanding the application of the CRISPR-Cas9 antimicrobial to a broad-range of β-lactamases genes with high sequence diversity (Kim et al., 2016).

### Type VI System

CRISPR-Cas13a (previously known as CRISPR-C2c2) system is the most recently identified system which belongs to the class 2 type VI system (Shmakov et al., 2015). This system is characterized by a single Cas13a RNase which cleaves single-stranded RNA (ssRNA) molecules in the crRNA-guided manner (Abudayyeh et al., 2016). Notably, this system simultaneously exhibits promiscuous collateral ssRNA degradation when it executes target RNA cleavage (East-Seletsky et al., 2016). Thus, cell growth of the bacterial host will be restricted when the CRISPR-Cas13a system conducts cleavage of transcripts encoded from the invading phages. Recently, Kiga et al. developed a series of CRISPR-Cas13a-based antimicrobials which are capable of killing carbapenem-resistant *E. coli* and methicillin-resistant *S. aureus* (MRSA; Kiga et al., 2020). Unlike the CRISPR-Cas9 antimicrobials which only re-sensitize bacterial cells when the target AMR genes are located on the plasmid and further require conventional antibiotics to completely eradicate the bacterial cells, CRISPR-Cas13a antimicrobials display strong activities of bacterial killing regardless of the locations of their targets. For example, introduction of the Cas13a protein and a crRNA targeting the carbapenem-resistant gene *bla*_IMP-1_ *in vitro* led to a 2~3-log reduction in the number of recovered bacterial cells which carry the resistant gene either on the chromosome or plasmid (Kiga et al., 2020). In contrast, introduction of Cas9 and a crRNA led to a 3-log reduction in the number of bacterial cells when the *bla*_IMP-1_ gene is only located on the chromosome. The phenomenon of bacterial killing by targeting the AMR gene either on chromosome or plasmid was also demonstrated by the phage-delivered CRISPR-Cas13a system but not the CRISPR-Cas9 system (Kiga et al., 2020), indicating that the CRISPR-Cas13a system is expected to be superior to the Cas9 system for the broad antimicrobial applications because many clinically important AMR genes are encoded on plasmids (Buckner et al., 2018). The potential of CRISPR-Cas13a antimicrobials to improve host survival during bacterial infection was demonstrated using a *G. mellonella* larvae infection model.

## Challenges in the Delivery of Crispr-Cas Antimicrobials

### Phage-Based Delivery

Bacterial pathogens commonly possess rich phage populations which highly adept at injecting DNA into the host bacterial cells. Thus, phages are regarded as the most promising tool for the delivery of CRISPR-Cas antimicrobials. Despite increasing studies have shown the use of phage-based delivery of CRISPR-Cas antimicrobials to remove AMR plasmids or kill AMR pathogens, there are still some limitations in the therapeutic applications of CRISPR-Cas antimicrobials in terms of this phage-based delivery method. First, the size of phage capsid is found to be associated with the phage genome size (Hua et al., 2017). Thus, incorporating the large CRISPR-Cas elements into a phage genome might consequently impair the phage replication and assembly. To maintain phage viability, it is necessary to first delete the non-essential DNA fragments or use the CRISPR-Cas elements to replace the non-essential DNA fragments in the phage genome. However, given that most phages are not well characterized, additional steps to reveal the functions of phage genes are required before the deletion of the non-essential DNA fragments. Secondly, the host ranges of most phage species are narrow because phage absorption, the first step of phage infection, is mediated by the interaction between a phage receptor-binding protein and a specific receptor on the host cell membrane (Dupont et al., 2004; Chatterjee and Rothenberg, 2012). The presence of the interaction between phage and receptor proteins indicates that a specific phage might be required to treat a particular pathogen. Therefore, understanding the mechanisms underlying phage absorption and subsequently engineering the existing phages that have already shown a great capacity to efficiently deliver CRISPR-Cas antimicrobials might potentially broaden their host range. For instance, studies have revealed that the phage tail module plays an important role in phage absorption and modification of the phage tail fiber protein can lead to altered host specificity (Le et al., 2013; Pires et al., 2016). Another concern on phage delivery is that phage may deliver not only the necessary CRISPR-Cas elements, but also chromosomal segments from the host which serves for phage propagation into target cells, raising the safety issues of spreading virulence factor genes (Penadés et al., 2015; Pirnay et al., 2015). To prevent spreading high-risk virulent genes *via* phage-mediated transduction, these genes could be firstly removed from the host genome prior to phage propagation (Park et al., 2017).

### Conjugative Plasmid and Nanoparticle-Mediated Delivery

Alternative delivery means could also be explored in addition to the phage-based delivery. One of the alternative delivery vehicles is the conjugative plasmid which can transfer genetic elements between bacteria cells (Figure 2B; Rodrigues et al., 2019; Ruotsalainen et al., 2019). Different from the phage-mediated delivery which requires a specific receptor for its recognition, conjugation does not require receptors for the plasmid uptake. Thus, resistance against phage-based delivery owing to the emerged mutations in the receptors will not occur in the plasmid-based delivery (Pereira et al., 2021). However, some other issues, such as the narrow host range and low delivery efficiency, also exist in plasmid conjugation (Pursey et al., 2018). Another vehicle is using nanoparticles to directly deliver the Cas effectors and crRNA molecules into the target bacterial cells (Figure 2B). With the rapid development of nanotechnology, multiple nanoparticles, such as the cationic polymer-based nanoparticles and inorganic nanoparticles, have been readily accessible to transfer the necessary components of CRISPR-Cas systems (Lee et al., 2017; Rahimi et al., 2020). It was shown that a cationic polymer-based nanosized CRISPR complex which carries the Cas9 protein and crRNA can be successfully introduced into MRSA *in vitro* and is functional to execute bacterial killing by targeting the methicillin-resistant gene (Kang et al., 2017). However, exploitations of nanoparticles-based CRISPR-Cas delivery are still at the very preliminary stage, many questions remain unsolved, such as how to improve encapsulation rate and how to achieve efficient delivery into peculiar pathogens, such as *Mycobacterium tuberculosis* which contains unusually thick and highly impermeable cell walls (Chiaradia et al., 2017).

### Directing CRISPR-Cas Antimicrobials to Intracellular Pathogens

Bacterial pathogens are divided into extracellular and intracellular pathogens according to their sites of replication (Yan et al., 2021). Many human pathogens, such as *M. tuberculosis*, *Burkholderia* spp., and *S. enterica*, belong to intracellular pathogens which can reside in different host cells and are capable of escaping from CRISPR-Cas-mediated eradication. Therefore, in addition to establish delivery vehicles for CRISPR-Cas antimicrobials, how to transport them to target intracellular pathogens is another major challenge. Unfortunately, current attempts to develop CRISPR-Cas antimicrobials are mostly based on *in vitro* studies, efforts should be made to translocate CRISPR-Cas antimicrobials across plasma membranes of host cells. Given that the successful demonstrations of delivering phages into eukaryotic cells by liposomes, avirulent bacterial strains etc. (Broxmeyer et al., 2002; Nieth et al., 2015; Yan et al., 2021), it is noteworthy to know whether these approaches could also be employed to transport CRISPR-Cas-carrying phages, conjugative plasmids, and nanoparticles to eradicate intracellular pathogens.

## Emerged Resistance Against Crispr-Cas Antimicrobials

Although studies have shown the strong potency in bacterial killing using the CRISPR-Cas antimicrobials, there are still colonies survived by escaping genome targeting (Citorik et al., 2014; Gomaa et al., 2014). Several factors mainly contribute to the emerged resistance against CRISPR-Cas antimicrobials in the escaped colonies, such as the spontaneous mutations in the Cas genes or the target sequences, spacer excision owing to the homologous recombination between the repeats, presence of the anti-CRISPR (Acr) genes in the target host genomes, and repressed expression/activity of *cas* proteins. In a recent study, when the genomes and mini-CRISPRs isolated from escaped colonies from CRISPR-Cas-mediated genome targeting were sequenced, either mutations in the *cas* genes or the excision of spacers were identified (Xu et al., 2021a). Thus, future efforts to reduce the emerged resistance against CRISPR-Cas antimicrobials should focus on preventing these spontaneous mutations in *cas* genes or crRNA-expressing elements, such as modifying the repeat sequences to prevent their homologous recombination (Csörgő et al., 2020). Mutation in the target sequence, such as the wide-spread variants of AMR genes, represents another important factor that can cause resistance to CRISPR-Cas antimicrobials. Same as the group of ESBLs, many other families of antibiotic destructases are composed of a large collection of variants. For example, Tet(X) is a group of flavin monooxygenases that confer high-level tigecycline and eravacycline resistance in *E. coli* and *Acinetobacter* spp. (Chen et al., 2020). To ensure the precise targeting and efficient destruction of AMR genes, methods for the rapid and robust detection of specific AMR genes are required to facilitate the design of CRISPR-Cas antimicrobials (Cui et al., 2020).

Acrs are small proteins that can inactivate CRISPR-Cas immunity by interacting with the key components of CRISPR-Cas systems (Marino et al., 2020). These proteins are highly diverse and broadly present in prokaryotes, which can inactivate almost all the types of CRISPR-Cas systems (Davidson et al., 2020). For example, based on a collection of over 600 genomes of AMR *P. aeruginosa*, more than 30% of them were found containing at least one *acr* gene (van Belkum et al., 2015), largely restricting the antimicrobial applications of CRISPR-Cas systems. Interestingly, a group of highly conserved anti-CRISPR-associated (*aca*) genes are frequently encoded at the 3'-end of *acr* gene regions (Marino et al., 2018). Their functions were identified as the natural repressors of the *acr* genes (Stanley et al., 2019). Therefore, to overcome Acrs, simultaneous overexpression of the Aca protein represents a promising “anti-anti-CRISPR” strategy to reactivate CRISPR-Cas antimicrobials. Feasibility of the “anti-anti-CRISPR” strategy has been indicated in a model strain *P. aeruginosa* PAO1 which was lysogenized by a recombinant DMS3m phage expressing an anti-CRISPR gene *acrIC1* (Csörgő et al., 2020). However, its robustness in the clinically relevant strains is compromised and remains further improvement (Xu et al., 2021a).

In addition to above-mentioned factors, it was shown that disruption of two major quorum sensing (QS) systems *las* and *rhl* led to a decreased CRISPR-Cas activity in *P. aeruginosa* PA14 (Høyland-Kroghsbo et al., 2017). This indicates that cell-cell communications within a bacterial population would also affect the efficacy of CRISPR-Cas antimicrobials. Furthermore, CRISPR-Cas activities are known to be regulated by temperature. It was interestingly reported that low temperature enhances the activity of the type I-F CRISPR-Cas system while high temperature enhances the type II system (Xiang et al., 2017; Høyland-Kroghsbo et al., 2018). Therefore, QS, temperature, and possibly other physiologically relevant stimuli should also be taken into consideration in promoting the activity of CRISPR-Cas systems to minimize the occurrence of resistance against CRISPR-Cas antimicrobials.

## Future Perspectives

CRISPR-Cas antimicrobials display a number of potential advantages over the conventional antimicrobials. CRISPR-Cas systems are highly diversified which include at least 33 subtypes (Makarova et al., 2020), while current exploitations are still very preliminary with a focus on the common types, such as the type I-E and I-B, type II Cas9, and type VI Cas13a systems. We envision that more CRISPR-Cas types could be explored to achieve versatile antimicrobial applications. Once the challenges in delivery and targeting efficiency are overcome, we expect that CRISPR-Cas systems could be designed as “smart” antimicrobials to control the composition of gut microbiome by distinguishing pathogenic and beneficial bacteria, eradicate AMR pathogens, and prevent the spread of AMR genes in the future medical applications.

## Author Contributions

ZX conceptualized the mini-review topic. CD, HC, L-HZ, and ZX contributed to writing the mini-review and editing the final version. All authors contributed to the article and approved the submitted version.

## Conflict of Interest

The authors declare that the research was conducted in the absence of any commercial or financial relationships that could be construed as a potential conflict of interest.

## Publisher’s Note

All claims expressed in this article are solely those of the authors and do not necessarily represent those of their affiliated organizations, or those of the publisher, the editors and the reviewers. Any product that may be evaluated in this article, or claim that may be made by its manufacturer, is not guaranteed or endorsed by the publisher.
